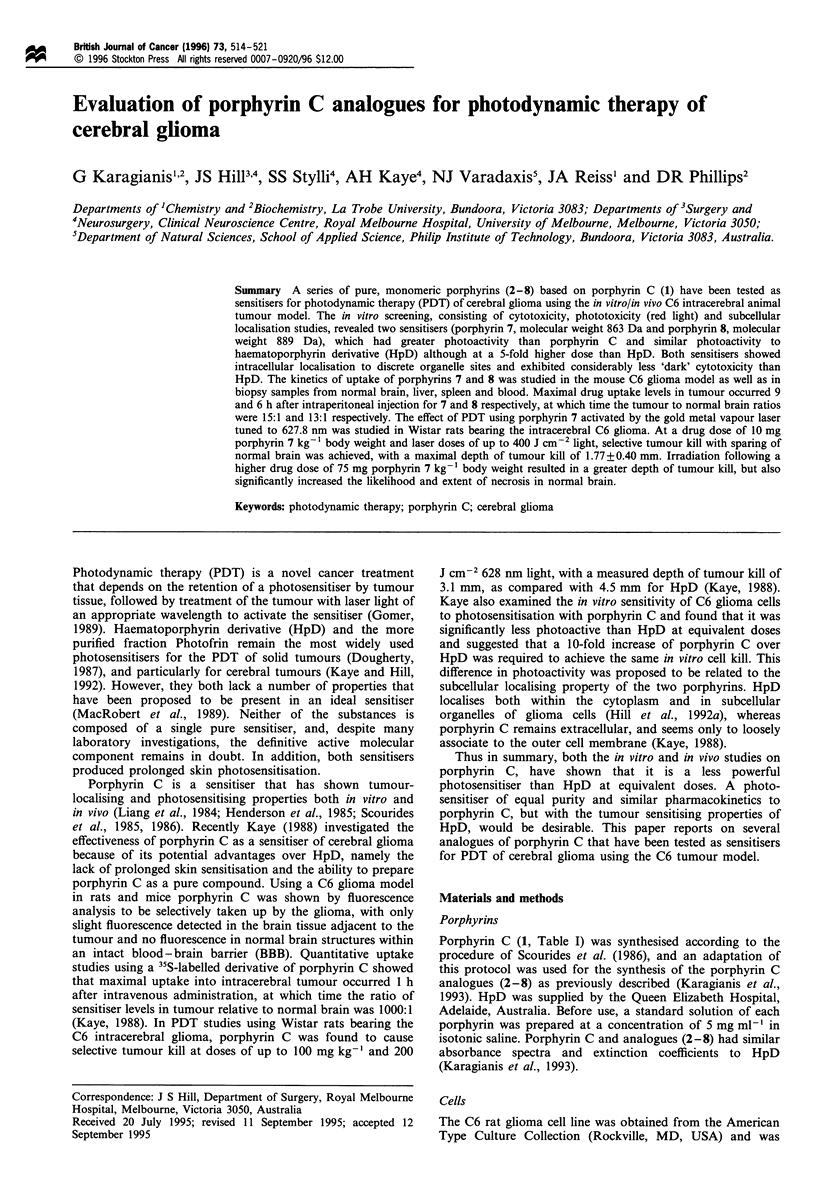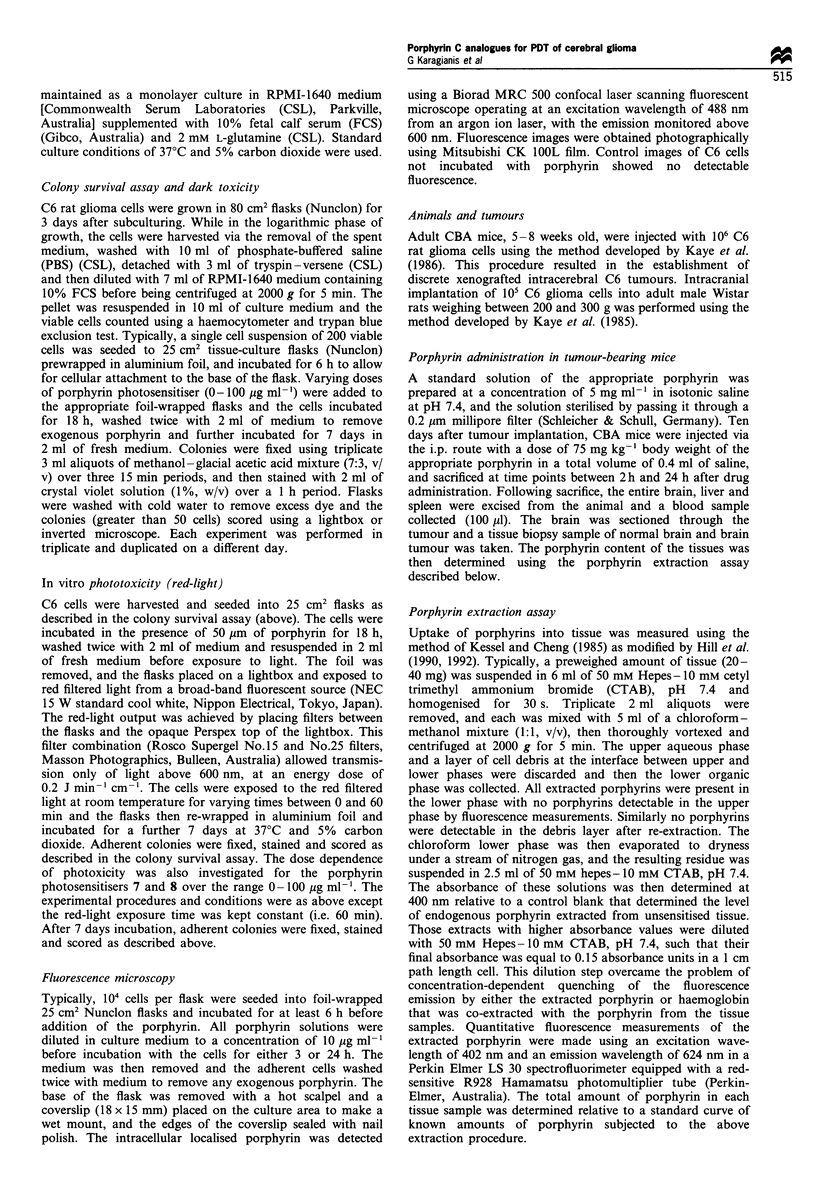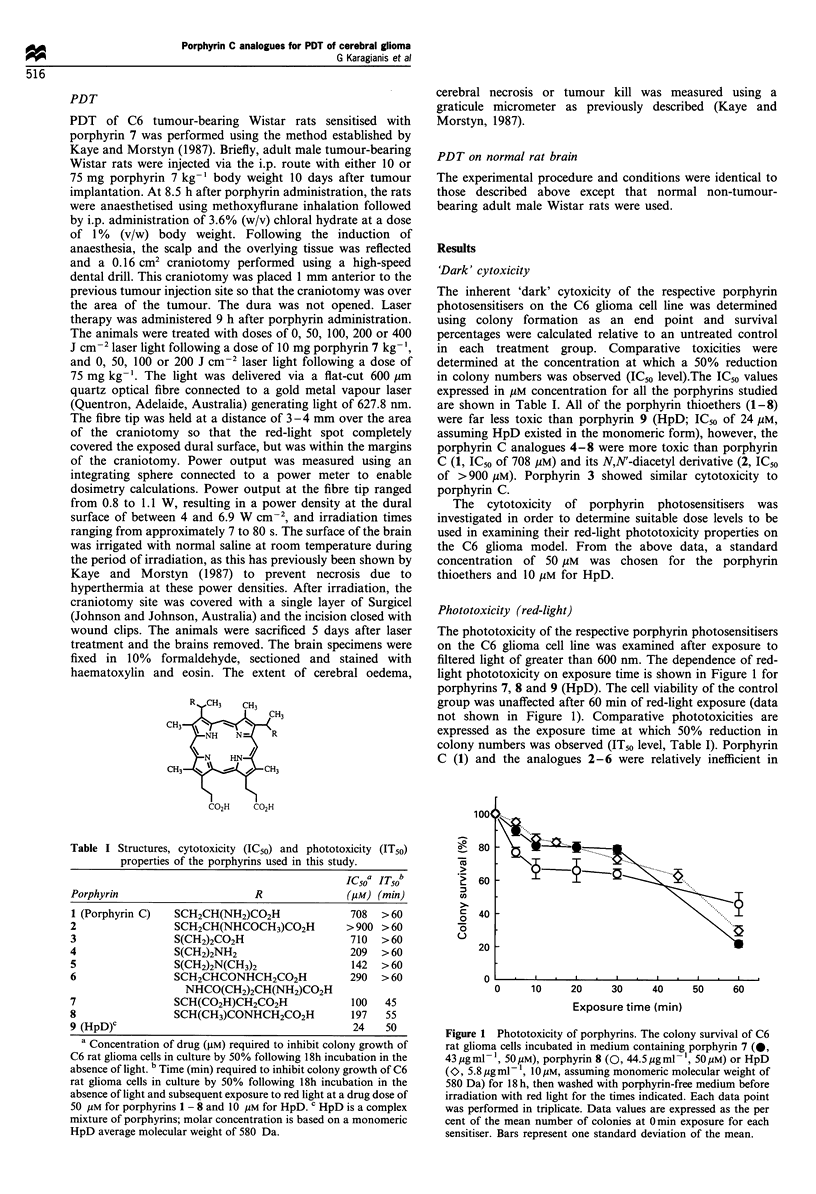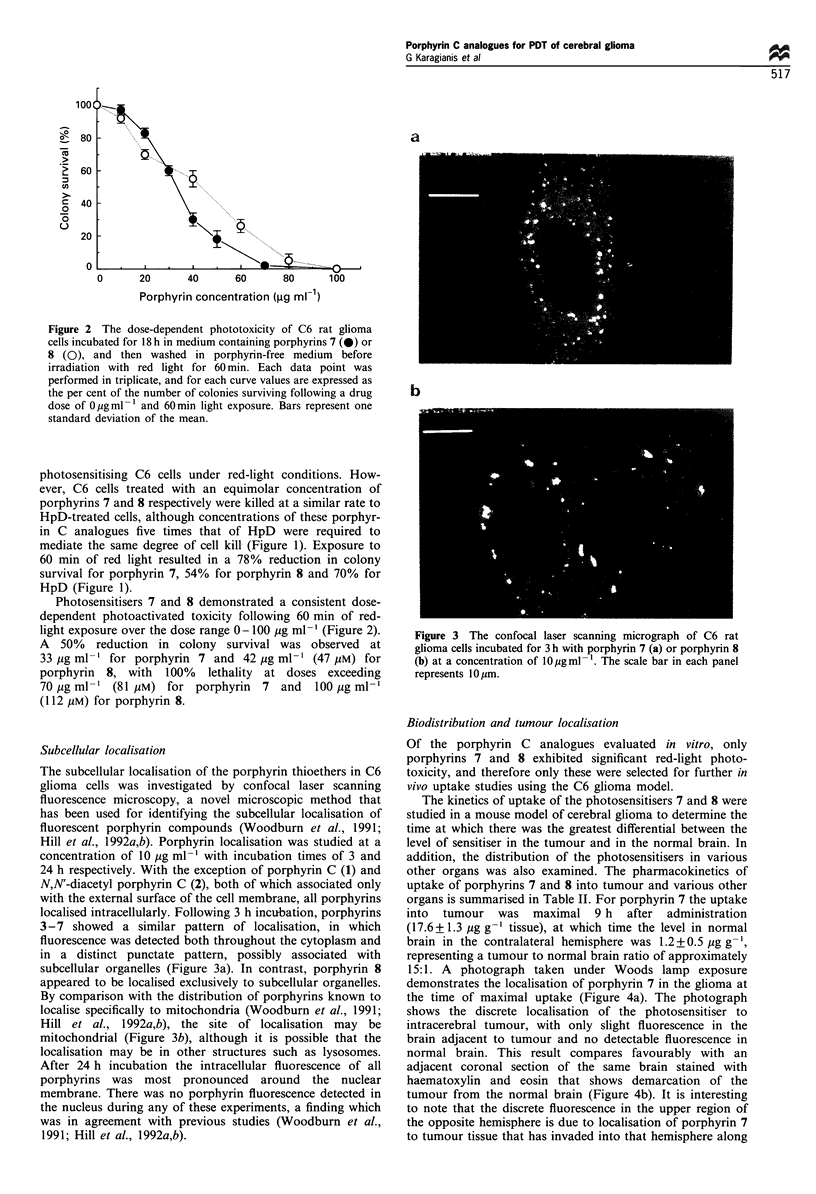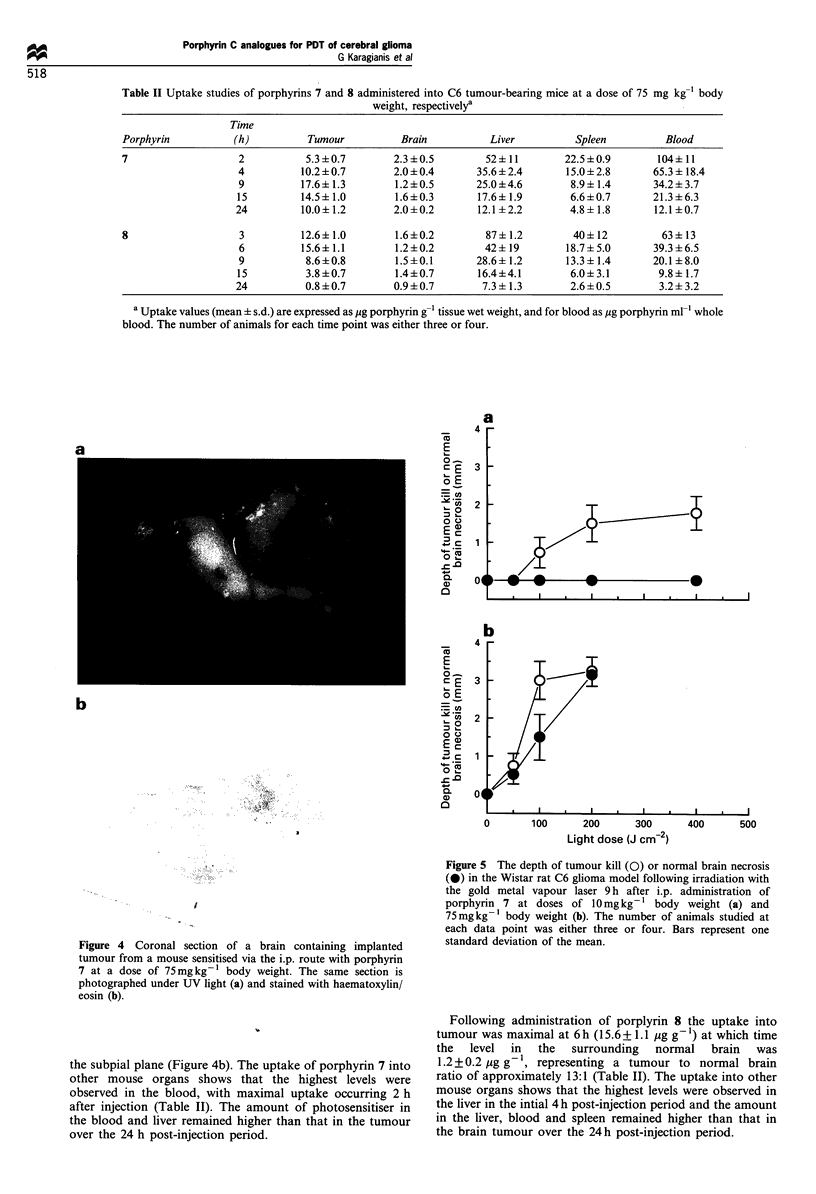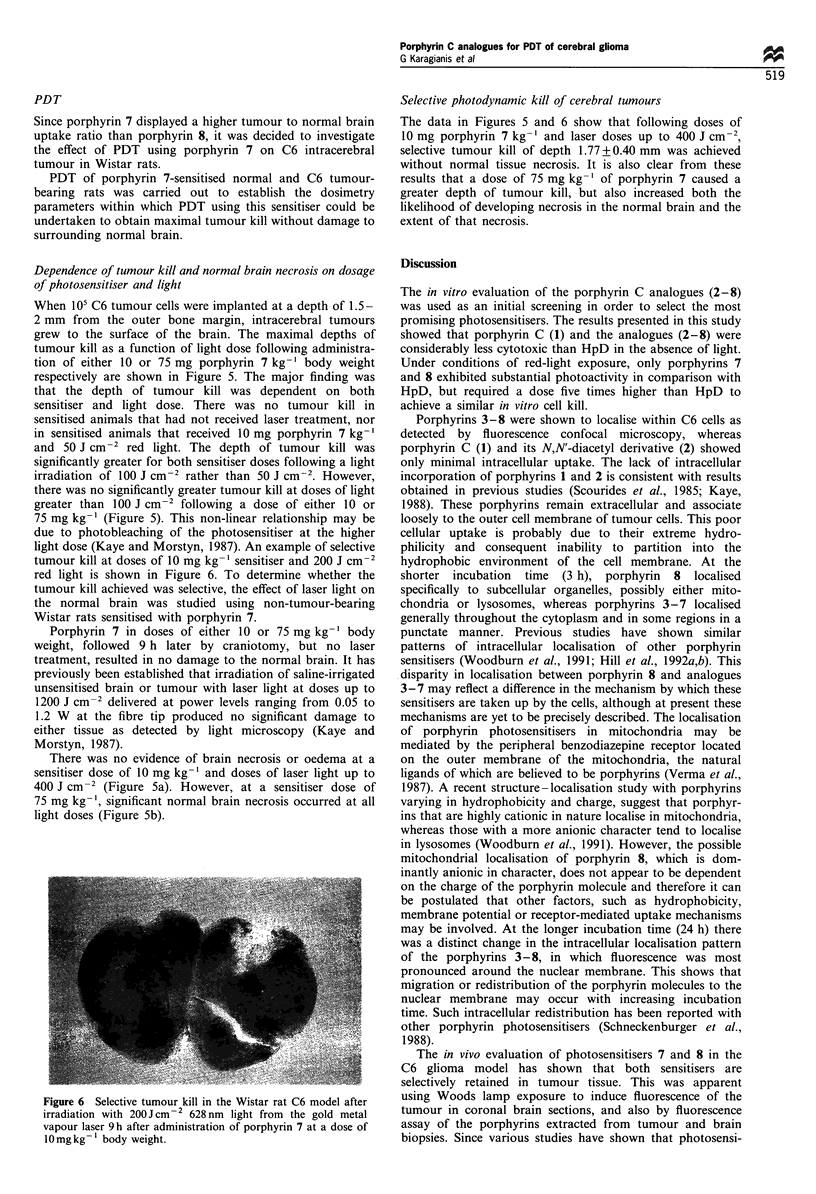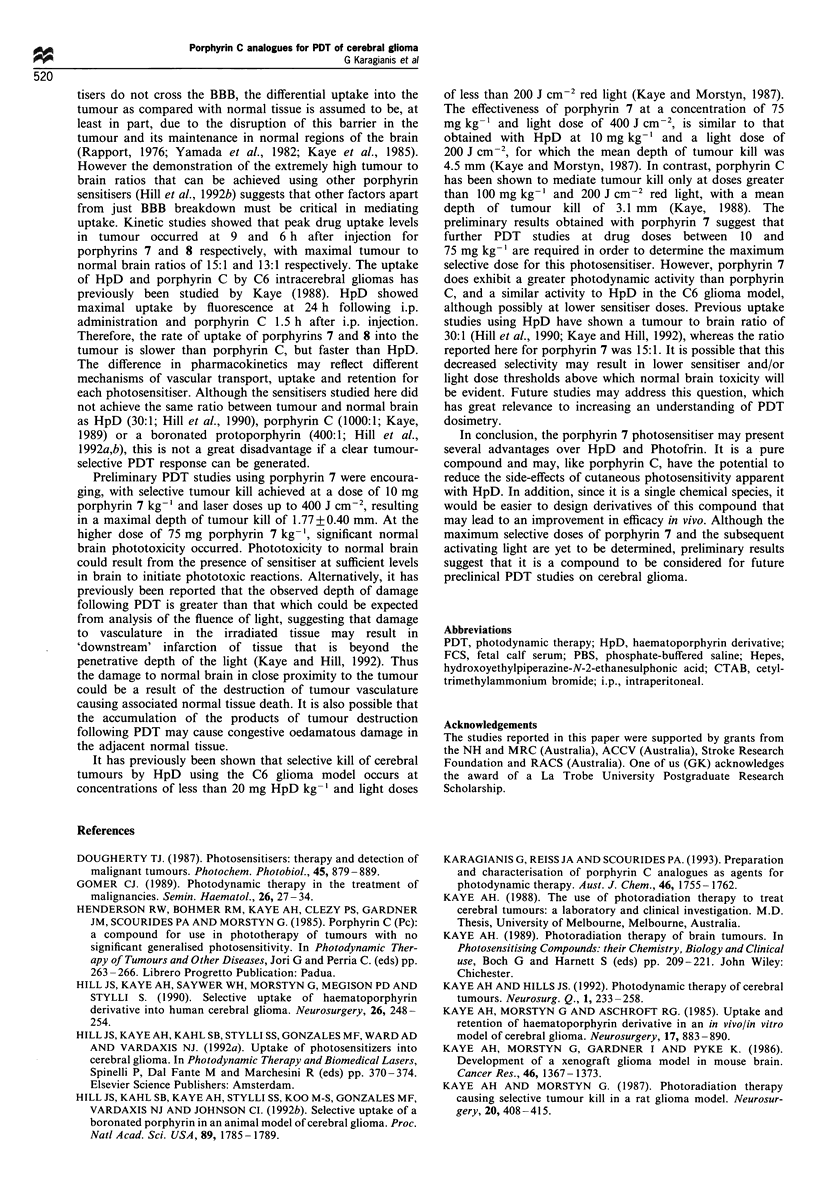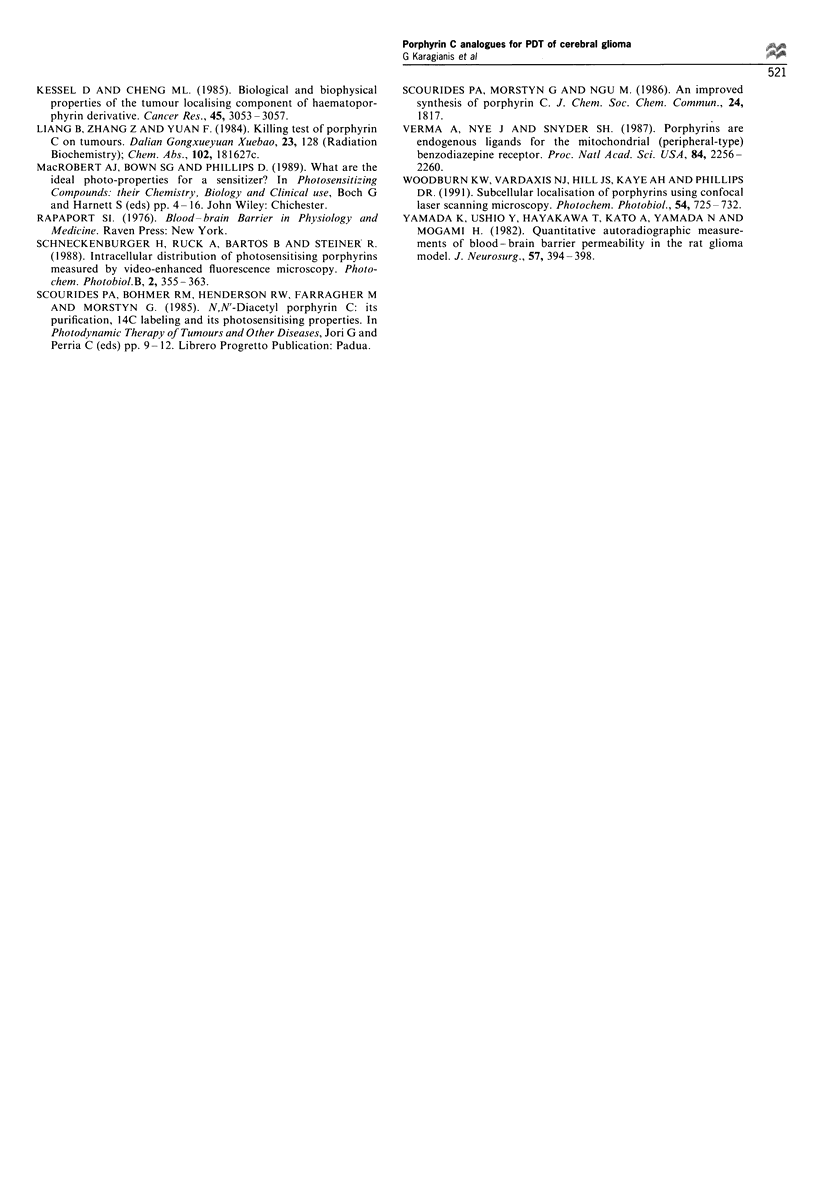# Evaluation of porphyrin C analogues for photodynamic therapy of cerebral glioma.

**DOI:** 10.1038/bjc.1996.89

**Published:** 1996-02

**Authors:** G. Karagianis, J. S. Hill, S. S. Stylli, A. H. Kaye, N. J. Varadaxis, J. A. Reiss, D. R. Phillips

**Affiliations:** Department of Chemistry, La Trobe University, Bundoora, Victoria, Australia.

## Abstract

**Images:**


British Journal of Cancer (1996) 73, 514-521
© 1996 Stockton Press All rights reserved 0007-0920/96 $12.00

### Evaluation of porphyrin C analogues for photodynamic therapy of cerebral glioma

G Karagianis[1,2], JS Hill[3,4], SS Stylli[4], AH Kaye[4], NJ Varadaxis[5], JA Reiss[1] and DR Phillips[2]

*Departments of [1]Chemistry and [2]Biochemistry, La Trobe University, Bundoora, Victoria 3083; Departments of [3]Surgery and [4]Neurosurgery, Clinical Neuroscience Centre, Royal Melbourne Hospital, University of Melbourne, Melbourne, Victoria 3050; [5]Department of Natural Sciences, School of Applied Science, Philip Institute of Technology, Bundoora, Victoria 3083, Australia.*

**Summary** A series of pure, monomeric porphyrins (**2–8**) based on porphyrin C (**1**) have been tested as sensitisers for photodynamic therapy (PDT) of cerebral glioma using the *in vitro/in vivo* C6 intracerebral animal tumour model. The *in vitro* screening, consisting of cytotoxicity, phototoxicity (red light) and subcellular localisation studies, revealed two sensitisers (porphyrin **7**, molecular weight 863 Da and porphyrin **8**, molecular weight 889 Da), which had greater photoactivity than porphyrin C and similar photoactivity to haematoporphyrin derivative (HpD) although at a 5-fold higher dose than HpD. Both sensitisers showed intracellular localisation to discrete organelle sites and exhibited considerably less 'dark' cytotoxicity than HpD. The kinetics of uptake of porphyrins **7** and **8** was studied in the mouse C6 glioma model as well as in biopsy samples from normal brain, liver, spleen and blood. Maximal drug uptake levels in tumour occurred 9 and 6 h after intraperitoneal injection for **7** and **8** respectively, at which time the tumour to normal brain ratios were 15:1 and 13:1 respectively. The effect of PDT using porphyrin **7** activated by the gold metal vapour laser tuned to 627.8 nm was studied in Wistar rats bearing the intracerebral C6 glioma. At a drug dose of 10 mg porphyrin **7** $kg^{-1}$ body weight and laser doses of up to 400 J $cm^{-2}$ light, selective tumour kill with sparing of normal brain was achieved, with a maximal depth of tumour kill of $1.77 \pm 0.40$ mm. Irradiation following a higher drug dose of 75 mg porphyrin **7** $kg^{-1}$ body weight resulted in a greater depth of tumour kill, but also significantly increased the likelihood and extent of necrosis in normal brain.

**Keywords:** photodynamic therapy; porphyrin C; cerebral glioma

Photodynamic therapy (PDT) is a novel cancer treatment that depends on the retention of a photosensitiser by tumour tissue, followed by treatment of the tumour with laser light of an appropriate wavelength to activate the sensitiser (Gomer, 1989). Haematoporphyrin derivative (HpD) and the more purified fraction Photofrin remain the most widely used photosensitisers for the PDT of solid tumours (Dougherty, 1987), and particularly for cerebral tumours (Kaye and Hill, 1992). However, they both lack a number of properties that have been proposed to be present in an ideal sensitiser (MacRobert *et al.*, 1989). Neither of the substances is composed of a single pure sensitiser, and, despite many laboratory investigations, the definitive active molecular component remains in doubt. In addition, both sensitisers produced prolonged skin photosensitisation.

Porphyrin C is a sensitiser that has shown tumour-localising and photosensitising properties both *in vitro* and *in vivo* (Liang *et al.*, 1984; Henderson *et al.*, 1985; Scourides *et al.*, 1985, 1986). Recently Kaye (1988) investigated the effectiveness of porphyrin C as a sensitiser of cerebral glioma because of its potential advantages over HpD, namely the lack of prolonged skin sensitisation and the ability to prepare porphyrin C as a pure compound. Using a C6 glioma model in rats and mice porphyrin C was shown by fluorescence analysis to be selectively taken up by the glioma, with only slight fluorescence detected in the brain tissue adjacent to the tumour and no fluorescence in normal brain structures within an intact blood–brain barrier (BBB). Quantitative uptake studies using a $^{35}S$-labelled derivative of porphyrin C showed that maximal uptake into intracerebral tumour occurred 1 h after intravenous administration, at which time the ratio of sensitiser levels in tumour relative to normal brain was 1000:1 (Kaye, 1988). In PDT studies using Wistar rats bearing the C6 intracerebral glioma, porphyrin C was found to cause selective tumour kill at doses of up to 100 mg $kg^{-1}$ and 200 J $cm^{-2}$ 628 nm light, with a measured depth of tumour kill of 3.1 mm, as compared with 4.5 mm for HpD (Kaye, 1988). Kaye also examined the *in vitro* sensitivity of C6 glioma cells to photosensitisation with porphyrin C and found that it was significantly less photoactive than HpD at equivalent doses and suggested that a 10-fold increase of porphyrin C over HpD was required to achieve the same *in vitro* cell kill. This difference in photoactivity was proposed to be related to the subcellular localising property of the two porphyrins. HpD localises both within the cytoplasm and in subcellular organelles of glioma cells (Hill *et al.*, 1992*a*), whereas porphyrin C remains extracellular, and seems only to loosely associate to the outer cell membrane (Kaye, 1988).

Thus in summary, both the *in vitro* and *in vivo* studies on porphyrin C, have shown that it is a less powerful photosensitiser than HpD at equivalent doses. A photosensitiser of equal purity and similar pharmacokinetics to porphyrin C, but with the tumour sensitising properties of HpD, would be desirable. This paper reports on several analogues of porphyrin C that have been tested as sensitisers for PDT of cerebral glioma using the C6 tumour model.

### Materials and methods

### *Porphyrins*

Porphyrin C (**1**, Table I) was synthesised according to the procedure of Scourides *et al.* (1986), and an adaptation of this protocol was used for the synthesis of the porphyrin C analogues (**2–8**) as previously described (Karagianis *et al.*, 1993). HpD was supplied by the Queen Elizabeth Hospital, Adelaide, Australia. Before use, a standard solution of each porphyrin was prepared at a concentration of 5 mg $ml^{-1}$ in isotonic saline. Porphyrin C and analogues (**2–8**) had similar absorbance spectra and extinction coefficients to HpD (Karagianis *et al.*, 1993).

### *Cells*

The C6 rat glioma cell line was obtained from the American Type Culture Collection (Rockville, MD, USA) and was

Correspondence: J S Hill, Department of Surgery, Royal Melbourne Hospital, Melbourne, Victoria 3050, Australia
Received 20 July 1995; revised 11 September 1995; accepted 12 September 1995

maintained as a monolayer culture in RPMI-1640 medium [Commonwealth Serum Laboratories (CSL), Parkville, Australia] supplemented with 10% fetal calf serum (FCS) (Gibco, Australia) and 2 mM L-glutamine (CSL). Standard culture conditions of 37°C and 5% carbon dioxide were used.

*Colony survival assay and dark toxicity*

C6 rat glioma cells were grown in 80 $cm^2$ flasks (Nunclon) for 3 days after subculturing. While in the logarithmic phase of growth, the cells were harvested via the removal of the spent medium, washed with 10 ml of phosphate-buffered saline (PBS) (CSL), detached with 3 ml of tryspin–versene (CSL) and then diluted with 7 ml of RPMI-1640 medium containing 10% FCS before being centrifuged at 2000 ***g*** for 5 min. The pellet was resuspended in 10 ml of culture medium and the viable cells counted using a haemocytometer and trypan blue exclusion test. Typically, a single cell suspension of 200 viable cells was seeded to 25 $cm^2$ tissue-culture flasks (Nunclon) prewrapped in aluminium foil, and incubated for 6 h to allow for cellular attachment to the base of the flask. Varying doses of porphyrin photosensitiser (0–100 $\mu g\ ml^{-1}$) were added to the appropriate foil-wrapped flasks and the cells incubated for 18 h, washed twice with 2 ml of medium to remove exogenous porphyrin and further incubated for 7 days in 2 ml of fresh medium. Colonies were fixed using triplicate 3 ml aliquots of methanol–glacial acetic acid mixture (7:3, v/v) over three 15 min periods, and then stained with 2 ml of crystal violet solution (1%, w/v) over a 1 h period. Flasks were washed with cold water to remove excess dye and the colonies (greater than 50 cells) scored using a lightbox or inverted microscope. Each experiment was performed in triplicate and duplicated on a different day.

In vitro *phototoxicity (red-light)*

C6 cells were harvested and seeded into 25 $cm^2$ flasks as described in the colony survival assay (above). The cells were incubated in the presence of 50 $\mu m$ of porphyrin for 18 h, washed twice with 2 ml of medium and resuspended in 2 ml of fresh medium before exposure to light. The foil was removed, and the flasks placed on a lightbox and exposed to red filtered light from a broad-band fluorescent source (NEC 15 W standard cool white, Nippon Electrical, Tokyo, Japan). The red-light output was achieved by placing filters between the flasks and the opaque Perspex top of the lightbox. This filter combination (Rosco Supergel No.15 and No.25 filters, Masson Photographics, Bulleen, Australia) allowed transmission only of light above 600 nm, at an energy dose of 0.2 J $min^{-1}$ $cm^{-1}$. The cells were exposed to the red filtered light at room temperature for varying times between 0 and 60 min and the flasks then re-wrapped in aluminium foil and incubated for a further 7 days at 37°C and 5% carbon dioxide. Adherent colonies were fixed, stained and scored as described in the colony survival assay. The dose dependence of photoxicity was also investigated for the porphyrin photosensitisers **7** and **8** over the range 0–100 $\mu g\ ml^{-1}$. The experimental procedures and conditions were as above except the red-light exposure time was kept constant (i.e. 60 min). After 7 days incubation, adherent colonies were fixed, stained and scored as described above.

*Fluorescence microscopy*

Typically, $10^4$ cells per flask were seeded into foil-wrapped 25 $cm^2$ Nunclon flasks and incubated for at least 6 h before addition of the porphyrin. All porphyrin solutions were diluted in culture medium to a concentration of 10 $\mu g\ ml^{-1}$ before incubation with the cells for either 3 or 24 h. The medium was then removed and the adherent cells washed twice with medium to remove any exogenous porphyrin. The base of the flask was removed with a hot scalpel and a coverslip (18 × 15 mm) placed on the culture area to make a wet mount, and the edges of the coverslip sealed with nail polish. The intracellular localised porphyrin was detected using a Biorad MRC 500 confocal laser scanning fluorescent microscope operating at an excitation wavelength of 488 nm from an argon ion laser, with the emission monitored above 600 nm. Fluorescence images were obtained photographically using Mitsubishi CK 100L film. Control images of C6 cells not incubated with porphyrin showed no detectable fluorescence.

*Animals and tumours*

Adult CBA mice, 5–8 weeks old, were injected with $10^6$ C6 rat glioma cells using the method developed by Kaye *et al.* (1986). This procedure resulted in the establishment of discrete xenografted intracerebral C6 tumours. Intracranial implantation of $10^5$ C6 glioma cells into adult male Wistar rats weighing between 200 and 300 g was performed using the method developed by Kaye *et al.* (1985).

*Porphyrin administration in tumour-bearing mice*

A standard solution of the appropriate porphyrin was prepared at a concentration of 5 mg $ml^{-1}$ in isotonic saline at pH 7.4, and the solution sterilised by passing it through a 0.2 $\mu m$ millipore filter (Schleicher & Schull, Germany). Ten days after tumour implantation, CBA mice were injected via the i.p. route with a dose of 75 mg $kg^{-1}$ body weight of the appropriate porphyrin in a total volume of 0.4 ml of saline, and sacrificed at time points between 2 h and 24 h after drug administration. Following sacrifice, the entire brain, liver and spleen were excised from the animal and a blood sample collected (100 $\mu l$). The brain was sectioned through the tumour and a tissue biopsy sample of normal brain and brain tumour was taken. The porphyrin content of the tissues was then determined using the porphyrin extraction assay described below.

*Porphyrin extraction assay*

Uptake of porphyrins into tissue was measured using the method of Kessel and Cheng (1985) as modified by Hill *et al.* (1990, 1992). Typically, a preweighed amount of tissue (20–40 mg) was suspended in 6 ml of 50 mM Hepes–10 mM cetyl trimethyl ammonium bromide (CTAB), pH 7.4 and homogenised for 30 s. Triplicate 2 ml aliquots were removed, and each was mixed with 5 ml of a chloroform–methanol mixture (1:1, v/v), then thoroughly vortexed and centrifuged at 2000 ***g*** for 5 min. The upper aqueous phase and a layer of cell debris at the interface between upper and lower phases were discarded and then the lower organic phase was collected. All extracted porphyrins were present in the lower phase with no porphyrins detectable in the upper phase by fluorescence measurements. Similarly no porphyrins were detectable in the debris layer after re-extraction. The chloroform lower phase was then evaporated to dryness under a stream of nitrogen gas, and the resulting residue was suspended in 2.5 ml of 50 mM hepes–10 mM CTAB, pH 7.4. The absorbance of these solutions was then determined at 400 nm relative to a control blank that determined the level of endogenous porphyrin extracted from unsensitised tissue. Those extracts with higher absorbance values were diluted with 50 mM Hepes–10 mM CTAB, pH 7.4, such that their final absorbance was equal to 0.15 absorbance units in a 1 cm path length cell. This dilution step overcame the problem of concentration-dependent quenching of the fluorescence emission by either the extracted porphyrin or haemoglobin that was co-extracted with the porphyrin from the tissue samples. Quantitative fluorescence measurements of the extracted porphyrin were made using an excitation wavelength of 402 nm and an emission wavelength of 624 nm in a Perkin Elmer LS 30 spectrofluorimeter equipped with a red-sensitive R928 Hamamatsu photomultiplier tube (Perkin-Elmer, Australia). The total amount of porphyrin in each tissue sample was determined relative to a standard curve of known amounts of porphyrin subjected to the above extraction procedure.

*PDT*

PDT of C6 tumour-bearing Wistar rats sensitised with porphyrin **7** was performed using the method established by Kaye and Morstyn (1987). Briefly, adult male tumour-bearing Wistar rats were injected via the i.p. route with either 10 or 75 mg porphyrin 7 $kg^{-1}$ body weight 10 days after tumour implantation. At 8.5 h after porphyrin administration, the rats were anaesthetised using methoxyflurane inhalation followed by i.p. administration of 3.6% (w/v) chloral hydrate at a dose of 1% (v/w) body weight. Following the induction of anaesthesia, the scalp and the overlying tissue was reflected and a 0.16 $cm^2$ craniotomy performed using a high-speed dental drill. This craniotomy was placed 1 mm anterior to the previous tumour injection site so that the craniotomy was over the area of the tumour. The dura was not opened. Laser therapy was administered 9 h after porphyrin administration. The animals were treated with doses of 0, 50, 100, 200 or 400 J $cm^{-2}$ laser light following a dose of 10 mg porphyrin **7** $kg^{-1}$, and 0, 50, 100 or 200 J $cm^{-2}$ laser light following a dose of 75 mg $kg^{-1}$. The light was delivered via a flat-cut 600 $\mu m$ quartz optical fibre connected to a gold metal vapour laser (Quentron, Adelaide, Australia) generating light of 627.8 nm. The fibre tip was held at a distance of 3–4 mm over the area of the craniotomy so that the red-light spot completely covered the exposed dural surface, but was within the margins of the craniotomy. Power output was measured using an integrating sphere connected to a power meter to enable dosimetry calculations. Power output at the fibre tip ranged from 0.8 to 1.1 W, resulting in a power density at the dural surface of between 4 and 6.9 W $cm^{-2}$, and irradiation times ranging from approximately 7 to 80 s. The surface of the brain was irrigated with normal saline at room temperature during the period of irradiation, as this has previously been shown by Kaye and Morstyn (1987) to prevent necrosis due to hyperthermia at these power densities. After irradiation, the craniotomy site was covered with a single layer of Surgicel (Johnson and Johnson, Australia) and the incision closed with wound clips. The animals were sacrificed 5 days after laser treatment and the brains removed. The brain specimens were fixed in 10% formaldehyde, sectioned and stained with haematoxylin and eosin. The extent of cerebral oedema, cerebral necrosis or tumour kill was measured using a graticule micrometer as previously described (Kaye and Morstyn, 1987).

*PDT on normal rat brain*

The experimental procedure and conditions were identical to those described above except that normal non-tumour-bearing adult male Wistar rats were used.

### Results

*'Dark' cytoxicity*

The inherent 'dark' cytoxicity of the respective porphyrin photosensitisers on the C6 glioma cell line was determined using colony formation as an end point and survival percentages were calculated relative to an untreated control in each treatment group. Comparative toxicities were determined at the concentration at which a 50% reduction in colony numbers was observed ($IC_{50}$ level).The $IC_{50}$ values expressed in $\mu$M concentration for all the porphyrins studied are shown in Table I. All of the porphyrin thioethers (**1–8**) were far less toxic than porphyrin **9** (HpD; $IC_{50}$ of 24 $\mu$M, assuming HpD existed in the monomeric form), however, the porphyrin C analogues **4–8** were more toxic than porphyrin C (**1**, $IC_{50}$ of 708 $\mu$M) and its *N,N'*-diacetyl derivative (**2**, $IC_{50}$ of >900 $\mu$M). Porphyrin **3** showed similar cytotoxicity to porphyrin C.

The cytotoxicity of porphyrin photosensitisers was investigated in order to determine suitable dose levels to be used in examining their red-light phototoxicity properties on the C6 glioma model. From the above data, a standard concentration of 50 $\mu$M was chosen for the porphyrin thioethers and 10 $\mu$M for HpD.

*Phototoxicity (red-light)*

The phototoxicity of the respective porphyrin photosensitisers on the C6 glioma cell line was examined after exposure to filtered light of greater than 600 nm. The dependence of red-light phototoxicity on exposure time is shown in Figure 1 for porphyrins **7**, **8** and **9** (HpD). The cell viability of the control group was unaffected after 60 min of red-light exposure (data not shown in Figure 1). Comparative phototoxicities are expressed as the exposure time at which 50% reduction in colony numbers was observed ($IT_{50}$ level, Table I). Porphyrin C (**1**) and the analogues **2–6** were relatively inefficient in

**Table I** Structures, cytotoxicity ($IC_{50}$) and phototoxicity ($IT_{50}$) properties of the porphyrins used in this study.

| *Porphyrin* | *R* | *$IC_{50}$[a] ($\mu$M)* | *$IT_{50}$[b] (min)* |
|---|---|---|---|
| **1** (Porphyrin C) | $SCH_2CH(NH_2)CO_2H$ | 708 | >60 |
| **2** | $SCH_2CH(NHCOCH_3)CO_2H$ | >900 | >60 |
| **3** | $S(CH_2)_2CO_2H$ | 710 | >60 |
| **4** | $S(CH_2)_2NH_2$ | 209 | >60 |
| **5** | $S(CH_2)_2N(CH_3)_2$ | 142 | >60 |
| **6** | $SCH_2CHCONHCH_2CO_2H$ $NHCO(CH_2)_2CH(NH_2)CO_2H$ | 290 | >60 |
| **7** | $SCH(CO_2H)CH_2CO_2H$ | 100 | 45 |
| **8** | $SCH(CH_3)CONHCH_2CO_2H$ | 197 | 55 |
| **9** (HpD)[c] | | 24 | 50 |

[a] Concentration of drug ($\mu$M) required to inhibit colony growth of C6 rat glioma cells in culture by 50% following 18h incubation in the absence of light. [b] Time (min) required to inhibit colony growth of C6 rat glioma cells in culture by 50% following 18h incubation in the absence of light and subsequent exposure to red light at a drug dose of 50 $\mu$M for porphyrins **1 – 8** and 10 $\mu$M for HpD. [c] HpD is a complex mixture of porphyrins; molar concentration is based on a monomeric HpD average molecular weight of 580 Da.

**Figure 1** Phototoxicity of porphyrins. The colony survival of C6 rat glioma cells incubated in medium containing porphyrin **7** (●, 43 $\mu g\,ml^{-1}$, 50 $\mu$M), porphyrin **8** (○, 44.5 $\mu g\,ml^{-1}$, 50 $\mu$M) or HpD (◇, 5.8 $\mu g\,ml^{-1}$, 10 $\mu$M, assuming monomeric molecular weight of 580 Da) for 18 h, then washed with porphyrin-free medium before irradiation with red light for the times indicated. Each data point was performed in triplicate. Data values are expressed as the per cent of the mean number of colonies at 0 min exposure for each sensitiser. Bars represent one standard deviation of the mean.

**Figure 2** The dose-dependent phototoxicity of C6 rat glioma cells incubated for 18 h in medium containing porphyrins **7** (●) or **8** (○), and then washed in porphyrin-free medium before irradiation with red light for 60 min. Each data point was performed in triplicate, and for each curve values are expressed as the per cent of the number of colonies surviving following a drug dose of 0 $\mu$g ml$^{-1}$ and 60 min light exposure. Bars represent one standard deviation of the mean.

photosensitising C6 cells under red-light conditions. However, C6 cells treated with an equimolar concentration of porphyrins **7** and **8** respectively were killed at a similar rate to HpD-treated cells, although concentrations of these porphyrin C analogues five times that of HpD were required to mediate the same degree of cell kill (Figure 1). Exposure to 60 min of red light resulted in a 78% reduction in colony survival for porphyrin **7**, 54% for porphyrin **8** and 70% for HpD (Figure 1).

Photosensitisers **7** and **8** demonstrated a consistent dose-dependent photoactivated toxicity following 60 min of red-light exposure over the dose range 0–100 $\mu$g ml$^{-1}$ (Figure 2). A 50% reduction in colony survival was observed at 33 $\mu$g ml$^{-1}$ for porphyrin **7** and 42 $\mu$g ml$^{-1}$ (47 $\mu$M) for porphyrin **8**, with 100% lethality at doses exceeding 70 $\mu$g ml$^{-1}$ (81 $\mu$M) for porphyrin **7** and 100 $\mu$g ml$^{-1}$ (112 $\mu$M) for porphyrin **8**.

*Subcellular localisation*

The subcellular localisation of the porphyrin thioethers in C6 glioma cells was investigated by confocal laser scanning fluorescence microscopy, a novel microscopic method that has been used for identifying the subcellular localisation of fluorescent porphyrin compounds (Woodburn *et al.*, 1991; Hill *et al.*, 1992*a*,*b*). Porphyrin localisation was studied at a concentration of 10 $\mu$g ml$^{-1}$ with incubation times of 3 and 24 h respectively. With the exception of porphyrin C (**1**) and *N*,*N*′-diacetyl porphyrin C (**2**), both of which associated only with the external surface of the cell membrane, all porphyrins localised intracellularly. Following 3 h incubation, porphyrins **3**–**7** showed a similar pattern of localisation, in which fluorescence was detected both throughout the cytoplasm and in a distinct punctate pattern, possibly associated with subcellular organelles (Figure 3a). In contrast, porphyrin **8** appeared to be localised exclusively to subcellular organelles. By comparison with the distribution of porphyrins known to localise specifically to mitochondria (Woodburn *et al.*, 1991; Hill *et al.*, 1992*a*,*b*), the site of localisation may be mitochondrial (Figure 3*b*), although it is possible that the localisation may be in other structures such as lysosomes. After 24 h incubation the intracellular fluorescence of all porphyrins was most pronounced around the nuclear membrane. There was no porphyrin fluorescence detected in the nucleus during any of these experiments, a finding which was in agreement with previous studies (Woodburn *et al.*, 1991; Hill *et al.*, 1992*a*,*b*).

**Figure 3** The confocal laser scanning micrograph of C6 rat glioma cells incubated for 3 h with porphyrin **7** (**a**) or porphyrin **8** (**b**) at a concentration of 10 $\mu$g ml$^{-1}$. The scale bar in each panel represents 10 $\mu$m.

*Biodistribution and tumour localisation*

Of the porphyrin C analogues evaluated *in vitro*, only porphyrins **7** and **8** exhibited significant red-light phototoxicity, and therefore only these were selected for further *in vivo* uptake studies using the C6 glioma model.

The kinetics of uptake of the photosensitisers **7** and **8** were studied in a mouse model of cerebral glioma to determine the time at which there was the greatest differential between the level of sensitiser in the tumour and in the normal brain. In addition, the distribution of the photosensitisers in various other organs was also examined. The pharmacokinetics of uptake of porphyrins **7** and **8** into tumour and various other organs is summarised in Table II. For porphyrin **7** the uptake into tumour was maximal 9 h after administration (17.6 ± 1.3 $\mu$g g$^{-1}$ tissue), at which time the level in normal brain in the contralateral hemisphere was 1.2 ± 0.5 $\mu$g g$^{-1}$, representing a tumour to normal brain ratio of approximately 15:1. A photograph taken under Woods lamp exposure demonstrates the localisation of porphyrin **7** in the glioma at the time of maximal uptake (Figure 4a). The photograph shows the discrete localisation of the photosensitiser to intracerebral tumour, with only slight fluorescence in the brain adjacent to tumour and no detectable fluorescence in normal brain. This result compares favourably with an adjacent coronal section of the same brain stained with haematoxylin and eosin that shows demarcation of the tumour from the normal brain (Figure 4b). It is interesting to note that the discrete fluorescence in the upper region of the opposite hemisphere is due to localisation of porphyrin **7** to tumour tissue that has invaded into that hemisphere along

**Table II** Uptake studies of porphyrins **7** and **8** administered into C6 tumour-bearing mice at a dose of 75 mg $kg^{-1}$ body weight, respectively[a]

| *Porphyrin* | *Time (h)* | *Tumour* | *Brain* | *Liver* | *Spleen* | *Blood* |
|---|---|---|---|---|---|---|
| 7 | 2 | 5.3±0.7 | 2.3±0.5 | 52±11 | 22.5±0.9 | 104±11 |
| | 4 | 10.2±0.7 | 2.0±0.4 | 35.6±2.4 | 15.0±2.8 | 65.3±18.4 |
| | 9 | 17.6±1.3 | 1.2±0.5 | 25.0±4.6 | 8.9±1.4 | 34.2±3.7 |
| | 15 | 14.5±1.0 | 1.6±0.3 | 17.6±1.9 | 6.6±0.7 | 21.3±6.3 |
| | 24 | 10.0±1.2 | 2.0±0.2 | 12.1±2.2 | 4.8±1.8 | 12.1±0.7 |
| 8 | 3 | 12.6±1.0 | 1.6±0.2 | 87±1.2 | 40±12 | 63±13 |
| | 6 | 15.6±1.1 | 1.2±0.2 | 42±19 | 18.7±5.0 | 39.3±6.5 |
| | 9 | 8.6±0.8 | 1.5±0.1 | 28.6±1.2 | 13.3±1.4 | 20.1±8.0 |
| | 15 | 3.8±0.7 | 1.4±0.7 | 16.4±4.1 | 6.0±3.1 | 9.8±1.7 |
| | 24 | 0.8±0.7 | 0.9±0.7 | 7.3±1.3 | 2.6±0.5 | 3.2±3.2 |

[a] Uptake values (mean ± s.d.) are expressed as μg porphyrin $g^{-1}$ tissue wet weight, and for blood as μg porphyrin $ml^{-1}$ whole blood. The number of animals for each time point was either three or four.

**Figure 4** Coronal section of a brain containing implanted tumour from a mouse sensitised via the i.p. route with porphyrin **7** at a dose of 75 mg $kg^{-1}$ body weight. The same section is photographed under UV light (**a**) and stained with haematoxylin/eosin (**b**).

**Figure 5** The depth of tumour kill (○) or normal brain necrosis (●) in the Wistar rat C6 glioma model following irradiation with the gold metal vapour laser 9 h after i.p. administration of porphyrin **7** at doses of 10 mg $kg^{-1}$ body weight (**a**) and 75 mg $kg^{-1}$ body weight (**b**). The number of animals studied at each data point was either three or four. Bars represent one standard deviation of the mean.

the subpial plane (Figure 4b). The uptake of porphyrin **7** into other mouse organs shows that the highest levels were observed in the blood, with maximal uptake occurring 2 h after injection (Table II). The amount of photosensitiser in the blood and liver remained higher than that in the tumour over the 24 h post-injection period.

Following administration of porplyrin **8** the uptake into tumour was maximal at 6 h (15.6 ± 1.1 μg $g^{-1}$) at which time the level in the surrounding normal brain was 1.2 ± 0.2 μg $g^{-1}$, representing a tumour to normal brain ratio of approximately 13:1 (Table II). The uptake into other mouse organs shows that the highest levels were observed in the liver in the intial 4 h post-injection period and the amount in the liver, blood and spleen remained higher than that in the brain tumour over the 24 h post-injection period.

*PDT*

Since porphyrin **7** displayed a higher tumour to normal brain uptake ratio than porphyrin **8**, it was decided to investigate the effect of PDT using porphyrin **7** on C6 intracerebral tumour in Wistar rats.

PDT of porphyrin **7**-sensitised normal and C6 tumour-bearing rats was carried out to establish the dosimetry parameters within which PDT using this sensitiser could be undertaken to obtain maximal tumour kill without damage to surrounding normal brain.

*Dependence of tumour kill and normal brain necrosis on dosage of photosensitiser and light*

When $10^5$ C6 tumour cells were implanted at a depth of 1.5–2 mm from the outer bone margin, intracerebral tumours grew to the surface of the brain. The maximal depths of tumour kill as a function of light dose following administration of either 10 or 75 mg porphyrin **7** $kg^{-1}$ body weight respectively are shown in Figure 5. The major finding was that the depth of tumour kill was dependent on both sensitiser and light dose. There was no tumour kill in sensitised animals that had not received laser treatment, nor in sensitised animals that received 10 mg porphyrin **7** $kg^{-1}$ and 50 J $cm^{-2}$ red light. The depth of tumour kill was significantly greater for both sensitiser doses following a light irradiation of 100 J $cm^{-2}$ rather than 50 J $cm^{-2}$. However, there was no significantly greater tumour kill at doses of light greater than 100 J $cm^{-2}$ following a dose of either 10 or 75 mg $kg^{-1}$ (Figure 5). This non-linear relationship may be due to photobleaching of the photosensitiser at the higher light dose (Kaye and Morstyn, 1987). An example of selective tumour kill at doses of 10 mg $kg^{-1}$ sensitiser and 200 J $cm^{-2}$ red light is shown in Figure 6. To determine whether the tumour kill achieved was selective, the effect of laser light on the normal brain was studied using non-tumour-bearing Wistar rats sensitised with porphyrin **7**.

Porphyrin **7** in doses of either 10 or 75 mg $kg^{-1}$ body weight, followed 9 h later by craniotomy, but no laser treatment, resulted in no damage to the normal brain. It has previously been established that irradiation of saline-irrigated unsensitised brain or tumour with laser light at doses up to 1200 J $cm^{-2}$ delivered at power levels ranging from 0.05 to 1.2 W at the fibre tip produced no significant damage to either tissue as detected by light microscopy (Kaye and Morstyn, 1987).

There was no evidence of brain necrosis or oedema at a sensitiser dose of 10 mg $kg^{-1}$ and doses of laser light up to 400 J $cm^{-2}$ (Figure 5a). However, at a sensitiser dose of 75 mg $kg^{-1}$, significant normal brain necrosis occurred at all light doses (Figure 5b).

**Figure 6** Selective tumour kill in the Wistar rat C6 model after irradiation with 200 J $cm^{-2}$ 628 nm light from the gold metal vapour laser 9 h after administration of porphyrin **7** at a dose of 10 mg $kg^{-1}$ body weight.

*Selective photodynamic kill of cerebral tumours*

The data in Figures 5 and 6 show that following doses of 10 mg porphyrin **7** $kg^{-1}$ and laser doses up to 400 J $cm^{-2}$, selective tumour kill of depth $1.77 \pm 0.40$ mm was achieved without normal tissue necrosis. It is also clear from these results that a dose of 75 mg $kg^{-1}$ of porphyrin **7** caused a greater depth of tumour kill, but also increased both the likelihood of developing necrosis in the normal brain and the extent of that necrosis.

### Discussion

The *in vitro* evaluation of the porphyrin C analogues (**2**–**8**) was used as an initial screening in order to select the most promising photosensitisers. The results presented in this study showed that porphyrin C (**1**) and the analogues (**2**–**8**) were considerably less cytotoxic than HpD in the absence of light. Under conditions of red-light exposure, only porphyrins **7** and **8** exhibited substantial photoactivity in comparison with HpD, but required a dose five times higher than HpD to achieve a similar *in vitro* cell kill.

Porphyrins **3**–**8** were shown to localise within C6 cells as detected by fluorescence confocal microscopy, whereas porphyrin C (**1**) and its *N,N'*-diacetyl derivative (**2**) showed only minimal intracellular uptake. The lack of intracellular incorporation of porphyrins **1** and **2** is consistent with results obtained in previous studies (Scourides *et al.*, 1985; Kaye, 1988). These porphyrins remain extracellular and associate loosely to the outer cell membrane of tumour cells. This poor cellular uptake is probably due to their extreme hydrophilicity and consequent inability to partition into the hydrophobic environment of the cell membrane. At the shorter incubation time (3 h), porphyrin **8** localised specifically to subcellular organelles, possibly either mitochondria or lysosomes, whereas porphyrins **3**–**7** localised generally throughout the cytoplasm and in some regions in a punctate manner. Previous studies have shown similar patterns of intracellular localisation of other porphyrin sensitisers (Woodburn *et al.*, 1991; Hill *et al.*, 1992*a*,*b*). This disparity in localisation between porphyrin **8** and analogues **3**–**7** may reflect a difference in the mechanism by which these sensitisers are taken up by the cells, although at present these mechanisms are yet to be precisely described. The localisation of porphyrin photosensitisers in mitochondria may be mediated by the peripheral benzodiazepine receptor located on the outer membrane of the mitochondria, the natural ligands of which are believed to be porphyrins (Verma *et al.*, 1987). A recent structure–localisation study with porphyrins varying in hydrophobicity and charge, suggest that porphyrins that are highly cationic in nature localise in mitochondria, whereas those with a more anionic character tend to localise in lysosomes (Woodburn *et al.*, 1991). However, the possible mitochondrial localisation of porphyrin **8**, which is dominantly anionic in character, does not appear to be dependent on the charge of the porphyrin molecule and therefore it can be postulated that other factors, such as hydrophobicity, membrane potential or receptor-mediated uptake mechanisms may be involved. At the longer incubation time (24 h) there was a distinct change in the intracellular localisation pattern of the porphyrins **3**–**8**, in which fluorescence was most pronounced around the nuclear membrane. This shows that migration or redistribution of the porphyrin molecules to the nuclear membrane may occur with increasing incubation time. Such intracellular redistribution has been reported with other porphyrin photosensitisers (Schneckenburger *et al.*, 1988).

The *in vivo* evaluation of photosensitisers **7** and **8** in the C6 glioma model has shown that both sensitisers are selectively retained in tumour tissue. This was apparent using Woods lamp exposure to induce fluorescence of the tumour in coronal brain sections, and also by fluorescence assay of the porphyrins extracted from tumour and brain biopsies. Since various studies have shown that photosensitisers do not cross the BBB, the differential uptake into the tumour as compared with normal tissue is assumed to be, at least in part, due to the disruption of this barrier in the tumour and its maintenance in normal regions of the brain (Rapport, 1976; Yamada *et al.*, 1982; Kaye *et al.*, 1985). However the demonstration of the extremely high tumour to brain ratios that can be achieved using other porphyrin sensitisers (Hill *et al.*, 1992*b*) suggests that other factors apart from just BBB breakdown must be critical in mediating uptake. Kinetic studies showed that peak drug uptake levels in tumour occurred at 9 and 6 h after injection for porphyrins **7** and **8** respectively, with maximal tumour to normal brain ratios of 15:1 and 13:1 respectively. The uptake of HpD and porphyrin C by C6 intracerebral gliomas has previously been studied by Kaye (1988). HpD showed maximal uptake by fluorescence at 24 h following i.p. administration and porphyrin C 1.5 h after i.p. injection. Therefore, the rate of uptake of porphyrins **7** and **8** into the tumour is slower than porphyrin C, but faster than HpD. The difference in pharmacokinetics may reflect different mechanisms of vascular transport, uptake and retention for each photosensitiser. Although the sensitisers studied here did not achieve the same ratio between tumour and normal brain as HpD (30:1; Hill *et al.*, 1990), porphyrin C (1000:1; Kaye, 1989) or a boronated protoporphyrin (400:1; Hill *et al.*, 1992*a*,*b*), this is not a great disadvantage if a clear tumour-selective PDT response can be generated.

Preliminary PDT studies using porphyrin **7** were encouraging, with selective tumour kill achieved at a dose of 10 mg porphyrin **7** $kg^{-1}$ and laser doses up to 400 J $cm^{-2}$, resulting in a maximal depth of tumour kill of $1.77 \pm 0.40$ mm. At the higher dose of 75 mg porphyrin **7** $kg^{-1}$, significant normal brain phototoxicity occurred. Phototoxicity to normal brain could result from the presence of sensitiser at sufficient levels in brain to initiate phototoxic reactions. Alternatively, it has previously been reported that the observed depth of damage following PDT is greater than that which could be expected from analysis of the fluence of light, suggesting that damage to vasculature in the irradiated tissue may result in 'downstream' infarction of tissue that is beyond the penetrative depth of the light (Kaye and Hill, 1992). Thus the damage to normal brain in close proximity to the tumour could be a result of the destruction of tumour vasculature causing associated normal tissue death. It is also possible that the accumulation of the products of tumour destruction following PDT may cause congestive oedamatous damage in the adjacent normal tissue.

It has previously been shown that selective kill of cerebral tumours by HpD using the C6 glioma model occurs at concentrations of less than 20 mg HpD $kg^{-1}$ and light doses of less than 200 J $cm^{-2}$ red light (Kaye and Morstyn, 1987). The effectiveness of porphyrin **7** at a concentration of 75 mg $kg^{-1}$ and light dose of 400 J $cm^{-2}$, is similar to that obtained with HpD at 10 mg $kg^{-1}$ and a light dose of 200 J $cm^{-2}$, for which the mean depth of tumour kill was 4.5 mm (Kaye and Morstyn, 1987). In contrast, porphyrin C has been shown to mediate tumour kill only at doses greater than 100 mg $kg^{-1}$ and 200 J $cm^{-2}$ red light, with a mean depth of tumour kill of 3.1 mm (Kaye, 1988). The preliminary results obtained with porphyrin **7** suggest that further PDT studies at drug doses between 10 and 75 mg $kg^{-1}$ are required in order to determine the maximum selective dose for this photosensitiser. However, porphyrin **7** does exhibit a greater photodynamic activity than porphyrin C, and a similar activity to HpD in the C6 glioma model, although possibly at lower sensitiser doses. Previous uptake studies using HpD have shown a tumour to brain ratio of 30:1 (Hill *et al.*, 1990; Kaye and Hill, 1992), whereas the ratio reported here for porphyrin **7** was 15:1. It is possible that this decreased selectivity may result in lower sensitiser and/or light dose thresholds above which normal brain toxicity will be evident. Future studies may address this question, which has great relevance to increasing an understanding of PDT dosimetry.

In conclusion, the porphyrin **7** photosensitiser may present several advantages over HpD and Photofrin. It is a pure compound and may, like porphyrin C, have the potential to reduce the side-effects of cutaneous photosensitivity apparent with HpD. In addition, since it is a single chemical species, it would be easier to design derivatives of this compound that may lead to an improvement in efficacy *in vivo*. Although the maximum selective doses of porphyrin **7** and the subsequent activating light are yet to be determined, preliminary results suggest that it is a compound to be considered for future preclinical PDT studies on cerebral glioma.

### Abbreviations

PDT, photodynamic therapy; HpD, haematoporphyrin derivative; FCS, fetal calf serum; PBS, phosphate-buffered saline; Hepes, hydroxoyethylpiperazine-*N*-2-ethanesulphonic acid; CTAB, cetyltrimethylammonium bromide; i.p., intraperitoneal.

### Acknowledgements

The studies reported in this paper were supported by grants from the NH and MRC (Australia), ACCV (Australia), Stroke Research Foundation and RACS (Australia). One of us (GK) acknowledges the award of a La Trobe University Postgraduate Research Scholarship.

### References

DOUGHERTY TJ. (1987). Photosensitisers: therapy and detection of malignant tumours. *Photochem. Photobiol.*, **45**, 879–889.

GOMER CJ. (1989). Photodynamic therapy in the treatment of malignancies. *Semin. Haematol.*, **26**, 27–34.

HENDERSON RW, BOHMER RM, KAYE AH, CLEZY PS, GARDNER JM, SCOURIDES PA AND MORSTYN G. (1985). Porphyrin C (Pc): a compound for use in phototherapy of tumours with no significant generalised photosensitivity. In *Photodynamic Therapy of Tumours and Other Diseases*, Jori G and Perria C. (eds) pp. 263–266. Librero Progretto Publication: Padua.

HILL JS, KAYE AH, SAYWER WH, MORSTYN G, MEGISON PD AND STYLLI S. (1990). Selective uptake of haematoporphyrin derivative into human cerebral glioma. *Neurosurgery*, **26**, 248–254.

HILL JS, KAYE AH, KAHL SB, STYLLI SS, GONZALES MF, WARD AD AND VARDAXIS NJ. (1992*a*). Uptake of photosensitizers into cerebral glioma. In *Photodynamic Therapy and Biomedical Lasers*, Spinelli P, Dal Fante M and Marchesini R (eds) pp. 370–374. Elsevier Science Publishers: Amsterdam.

HILL JS, KAHL SB, KAYE AH, STYLLI SS, KOO M-S, GONZALES MF, VARDAXIS NJ AND JOHNSON CI. (1992*b*). Selective uptake of a boronated porphyrin in an animal model of cerebral glioma. *Proc. Natl Acad. Sci. USA*, **89**, 1785–1789.

KARAGIANIS G, REISS JA AND SCOURIDES PA. (1993). Preparation and characterisation of porphyrin C analogues as agents for photodynamic therapy. *Aust. J. Chem.*, **46**, 1755–1762.

KAYE AH. (1988). The use of photoradiation therapy to treat cerebral tumours: a laboratory and clinical investigation. M.D. Thesis, University of Melbourne, Melbourne, Australia.

KAYE AH. (1989). Photoradiation therapy of brain tumours. In *Photosensitising Compounds: their Chemistry, Biology and Clinical use*, Boch G and Harnett S (eds) pp. 209–221. John Wiley: Chichester.

KAYE AH AND HILLS JS. (1992). Photodynamic therapy of cerebral tumours. *Neurosurg. Q.*, **1**, 233–258.

KAYE AH, MORSTYN G AND ASCHROFT RG. (1985). Uptake and retention of haematoporphyrin derivative in an *in vivo/in vitro* model of cerebral glioma. *Neurosurgery*, **17**, 883–890.

KAYE AH, MORSTYN G, GARDNER I AND PYKE K. (1986). Development of a xenograft glioma model in mouse brain. *Cancer Res.*, **46**, 1367–1373.

KAYE AH AND MORSTYN G. (1987). Photoradiation therapy causing selective tumour kill in a rat glioma model. *Neurosurgery*, **20**, 408–415.

KESSEL D AND CHENG ML. (1985). Biological and biophysical properties of the tumour localising component of haematoporphyrin derivative. *Cancer Res.*, **45,** 3053–3057.

LIANG B, ZHANG Z AND YUAN F. (1984). Killing test of porphyrin C on tumours. *Dalian Gongxueyuan Xuebao*, **23,** 128 (Radiation Biochemistry); *Chem. Abs.*, **102,** 181627c.

MacROBERT AJ, BOWN SG AND PHILLIPS D. (1989). What are the ideal photo-properties for a sensitizer? In *Photosensitizing Compounds: their Chemistry, Biology and Clinical use*, Boch G and Harnett S (eds) pp. 4–16. John Wiley: Chichester.

RAPAPORT SI. (1976). *Blood–brain Barrier in Physiology and Medicine*. Raven Press: New York.

SCHNECKENBURGER H, RUCK A, BARTOS B AND STEINER R. (1988). Intracellular distribution of photosensitising porphyrins measured by video-enhanced fluorescence microscopy. *Photochem. Photobiol.*B, **2,** 355–363.

SCOURIDES PA, BOHMER RM, HENDERSON RW, FARRAGHER M AND MORSTYN G. (1985). *N,N'*-Diacetyl porphyrin C: its purification, 14C labeling and its photosensitising properties. In *Photodynamic Therapy of Tumours and Other Diseases*, Jori G and Perria C (eds) pp. 9–12. Librero Progretto Publication: Padua.

SCOURIDES PA, MORSTYN G AND NGU M. (1986). An improved synthesis of porphyrin C. *J. Chem. Soc. Chem. Commun.*, **24,** 1817.

VERMA A, NYE J AND SNYDER SH. (1987). Porphyrins are endogenous ligands for the mitochondrial (peripheral-type) benzodiazepine receptor. *Proc. Natl Acad. Sci. USA*, **84,** 2256–2260.

WOODBURN KW, VARDAXIS NJ, HILL JS, KAYE AH AND PHILLIPS DR. (1991). Subcellular localisation of porphyrins using confocal laser scanning microscopy. *Photochem. Photobiol.*, **54,** 725–732.

YAMADA K, USHIO Y, HAYAKAWA T, KATO A, YAMADA N AND MOGAMI H. (1982). Quantitative autoradiographic measurements of blood–brain barrier permeability in the rat glioma model. *J. Neurosurg.*, **57,** 394–398.